# Feasibility of monitoring the resolution of acute pulmonary embolism with non-contrast-enhanced magnetic resonance imaging at one day, one week, one, three, and six months

**DOI:** 10.1177/02841851221122449

**Published:** 2022-09-14

**Authors:** Koshiar Medson, Eli Westerlund, Roberto Vargas Paris, Alexander Fyrdahl, Nina Vidovic, Sven Nyren, Peter Lindholm

**Affiliations:** 1 Department of Physiology and Pharmacology, 27106 Karolinska Institutet, Stockholm, Sweden; 2 Department of Imaging and Physiology, Karolinska University Hospital, Stockholm, Sweden; 3 Department of Clinical Sciences, 27106 Karolinska Institutet, Stockholm, Sweden; 4 Department of Internal medicine, Danderyd Hospital, Stockholm, Sweden; 5 Department of Molecular Medicine and Surgery, 27106 Karolinska Institutet, Stockholm, Sweden; 6 Department of Radiology, Mälarsjukhuset, Eskilstuna, Sweden; 7 Department of Emergency Medicine, University of California San Diego, San Diego, CA, USA

**Keywords:** Venous thromboembolism, pulmonary embolism, computed tomography pulmonary angiography, magnetic resonance imaging, steady-state free precession, unenhanced magnetic resonance imaging

## Abstract

**Background:**

Pulmonary embolism (PE) is a common cause of death with an incidence of approximately 1–2 cases per 1000 inhabitants in Europe and the United States. Treatment for PE is the administration of anticoagulants for at least three months.

**Purpose:**

To assess the feasibility of following the resolution rate of PE over time using repeated imaging with a non-contrast-enhanced magnetic resonance imaging (MRI) protocol.

**Material and Methods:**

Patients (n = 18) diagnosed with acute PE via computed tomography pulmonary angiography (CTPA) underwent non-contrast-enhanced MRI at two tertiary hospitals. The first MRI was performed within 36 h of CTPA, with follow-up at one week, one, three, and six months. The MRI sequence used was a non-contrast-enhanced standard two-dimensional steady-state free precession under free-breathing and without respiratory or cardiac gating. All MRI scans were then compared to the initial CTPA. The emboli were assessed visually for location and size, and clot burden was calculated using the Qanadli score.

**Results:**

MRI revealed complete resolution in seven cases at one week, in five cases at one month, and in three cases at three months. The most significant resolution of emboli occurred within the first few weeks, with only 10% of the diagnosed emboli persisting at the one-month examination.

**Conclusion:**

The use of MRI imparts the ability to visualize PE without radiation and thus allows multiple examinations to be made, for example in studies investigating the resolution of PE or the evaluation of drug effect in clinical trials.

## Introduction

Of the cardiovascular diseases, pulmonary embolism (PE) is the third most common cause of death and has an incidence of approximately 1–2 cases per 1000 inhabitants in Europe and the United States. In the USA alone, it is estimated that 60,000–100,000 deaths are related to venous thromboembolism (VTE) annually (1–4).

The diagnosis of PE is based on an assessment of clinical probability with the help of several clinical decision support (CDS) systems, but the definitive diagnosis is made by imaging (3). Today, the imaging modality of first choice is computed tomography pulmonary angiography (CTPA), which has a high sensitivity (83%) and specificity (96%) (5). CTPA exposes the patients to ionizing radiation, which is a concern for the development of malignancies (6).

Diagnosed cases of PE are treated with anticoagulants for at least three months. The length of treatment depends on several factors and is predetermined as described in the European Society of Cardiology (ESC) guidelines that were updated in 2019 (3). The recommended follow-up is at 3–6 months, and this decision is based currently on the patients’ symptoms, not imaging. In cases with symptoms such as dyspnea and/or functional limitation, the patient is investigated further with transthoracic echocardiography and a ventilation/perfusion (V/Q) scan (3). It is estimated that 4% will develop chronic PE and pulmonary hypertension after acute PE, although the etiology of this development is not completely understood (7).

In order to increase our understanding of the resolution process and its rate, we need an imaging modality that allows for serial examinations without exposing the patient to radiation. Several studies have tried to use magnetic resonance imaging (MRI) as an alternative to CTPA regarding the initial diagnosis of PE (8–11). However, many of them have used gadolinium-containing contrast agents together with respiratory and cardiac gating, which has rendered them technically challenging and thereby decreased their usability. We have therefore developed an MRI method that allows imaging of the pulmonary arteries within less than 10 min and without the need for a contrast agent, cardiac or respiratory gating, and is under free breathing. This in turn has allowed us to image the patients multiple times during this study and to visualize the resolution of the PE and its rate over time.

## Material and Methods

The present study was performed according to the Declaration of Helsinki and approved by the local Ethics Committee. Written informed consent was obtained from each patient before undergoing an MRI scan.

### Patients

Patients with a confirmed diagnosis of acute PE on CTPA underwent non-contrast-enhanced MRI. They were recruited from three tertiary hospitals and underwent MRI at only two of these, both of which had the same version/model of the scanner, a Siemens Magnetom Aera 1.5 T, in order to maintain consistency across the results. The CTPA scans were performed according to local protocols used at these tertiary hospitals.

The inclusion criteria were as follows: aged over 18 years; provided written informed consent; a confirmed diagnosis of acute PE on CTPA; and clinical parameters and the patient’s condition would allow for an additional examination as decided by the patient’s physician.

The exclusion criteria were as follows: contraindications for MRI; and more than 36 h past the diagnosis on CTPA.

### MRI

The MRI sequence used was a non-contrast-enhanced standard two-dimensional (2D) steady-state free precession (SSFP) under free-breathing and without respiratory or cardiac gating. The patients received no specific breathing instructions. Acquisition time was approximately 10 min for all three planes (axial = 3 min 50 s, sagittal and coronal = 2 min 52 s each). Five slices were obtained in each anatomical position in three orthogonal planes, generating stacks of approximately 500 images in each plane (axial = 600, sagittal = 450, and coronal = 450). The 1500 images were sorted by position, generating stacks with multiple images in various phases of the breathing and cardiac cycle. This MRI protocol has been used in three publications by our research group (12–14).

The first MRI was done within 36 h of the CTPA-confirmed diagnosis of PE, with a follow-up MRI examination after one week and one, three, and six months. We scheduled the MRI exams at 1 week ± 2 days and the exams at 1, 3, and 6 months ± 1 week.

### Analysis

There are multiple ways to analyze the data regarding the resolution of PE. In the present study, the following approach was used. First, we looked at the complete resolution of the emboli at the patient level. Second, we looked at the number of emboli at different artery levels (central, lobar, segmental) and the resolution rate at those levels. Finally, we calculated the clot burden according to the Qanadli score (15) to see the rate of change in clot burden over the study period.

The criteria for the diagnosis of PE were according to the PIOPED II study (16). The localization of each diagnosed emboli was determined at the following levels: central (pulmonary trunk until the lobar arteries); right upper/middle/lower lobe artery + segmental arteries; and left upper/middle/lower lobe artery + segmental arteries.

Each of the above levels were diagnosed independently, even when a single embolus extended through several of them. For example, if an embolus continued from lobar to two segmental arteries, it was described as three emboli at three different locations. We did not include the subsegmental level due to uncertainty of the diagnosis of PE at that point (5,12,17).

All the following MRI scans were then compared to the initial CTPA. The emboli on each subsequent MRI were graded as follows: 1 = emboli; X = undetermined; and 2 = no emboli. Two radiologists with a combined experience of six years as thoracic radiologists read all examinations, including the initial CTPA, and a consensus was reached in the case of any disagreements.

We also calculated the clot burden in these patients and the change over time using the Qanadli score (15). The same criteria were used for calculating the score for subsequent MRI scans as for the initial CTPA. The Qanadli scoring system divides each lung into 10 segments according to the following: upper lobes = three segments; lingula / middle lobe = two segments; lower lobes = five segments. An embolus in each segmental artery equals 1 point, and an embolus at the more proximal arteries gives points equal to the number of segmental arteries arising from them. There is also a weighting factor assigned to each embolus to add information about the residual perfusion. Finally, a percentage is calculated according to the following:

Σ(n⋅d)/40×100

Where *n* is the value of the proximal thrombus in the pulmonary arterial tree equal to the number of segmental branches arising distally (minimum = 1, maximum = 20), and *d* is the degree of obstruction (partial obstruction = 1, complete obstruction = 2) (15).

We also reviewed the patients’ electronic medical records (EMR) to follow their treatment and symptoms during the study.

## Results

Between September 2018 and March 2020, 18 patients (6 men, 12 women; age range = 30–77 years; mean age = 58 ± 15 years; median age = 60 years) were recruited to the study. Characteristics of the patients and relevant clinical information are summarized in Table 1.

**Table 1.** Clinical parameters for patients.

Patient No.	Age (years)	Sex	Reason for ER visit	Duration of symptoms	Malignancy	Sat	RR	HR	BP	Temperature (°C)	CRP	D-dimer
1	67	F	Dyspnea	<3 h	Breast cancer	98	18	117	120/71	37.7	X	X
2	30	M	Trauma	Low sat post op	No malignancy	94	24	93	139/90	37.7	24	X
3	77	M	Chest pain	5 days	No malignancy	95	16	70	120/80	38.0	72	3.8
4	77	F	Chest pain	<3 h	No malignancy	95	28	80	129/73	36.8	48	4.6
5	55	F	Dyspnea	1 week	No malignancy	100	16	80	140/90	37.9	100	X
6	56	F	Dyspnea	1 day	No malignancy	98	20	101	128/80	37.2	40	1.53
7	49	F	Dyspnea	Almost 1 year	Breast cancer in remission	99	16	80	140/90	37.1	3	1.57
8	69	F	Dyspnea	<3 h	Lung cancer	99	16	91	148/73	36.9	83	X
9	40	M	Chest pain	<3 h	Sarcoma	95	20	72	122/77	37.7	73	X
10	75	F	Dyspnea	2 months	Breast cancer in remission	99	16	84	123/70	36.6	3	X
11	51	M	Dyspnea	2 weeks	No malignancy	87	30	120	120/80	37.5	119	7.2
12	64	M	Dyspnea	<3 h	No malignancy	X	X	X	X	X	25	8.7
13	39	F	Dyspnea	<3 h	No malignancy	100	20	61	135/77	36.7	18	4.2
14	32	F	Back pain	2 days	No malignancy	X	X	X	X	X	31	2.34
15	65	F	Dyspnea	<3 h	No malignancy	100	16	77	173/80	36.8	5	6.4
16	71	F	Chest pain	1 week	Sigmoideum cancer	96	20	102	130/70	38.2	100	X
17	56	M	Dyspnea	2 weeks	No malignancy	92	20	105	128/80	38.0	184	3.6
18	70	F	Dyspnea	Few weeks	No malignancy	X	X	X	X	X	6	9.8

X = missing data in EMR.

BP, blood pressure; CRP, C Reactive Protein; EMR, Electronic medical records; ER, emergency room; HR, Heart rate; RR, Respiratory rate.

A total of 18 CTPA scans were obtained during the study period. With regard to the number of MRI scans made, one patient died before the one-month scheduled MRI due to terminal cancer and was therefore excluded, while another two examinations were missed at one-week post-CTPA, one at one month, two at three months, and two at six months. This was due to cancellation of appointments by the patients. Thus, the total number of MRI scans included in the study was 80. All exams are shown in Table 2.

**Table 2.** Timeline for CTPA and MRI exams.

Patient No.	Age (years)	Sex	CTPA			MRI 24 h			MRI 1 week			MRI 1 month			MRI 3 months			MRI 6 months		
			Central	Lobar	Seg	Central	Lobar	Seg	Central	Lobar	Seg	Central	Lobar	Seg	Central	Lobar	Seg	Central	Lobar	Seg
			Qanadli score			Qanadli score			Qanadli score			Qanadli score			Qanadli score			Qanadli score		
1	67	F	0	1	3	0	1	3	0	1	2	No scan			0	0	0	No scan		
			20%			20%			17,5%											
2	30	M	0	3	10	0	3	10	No scan			0	0	0	0	0	0	0	0	0
			30%			30%														
3	77	M	0	2	6	0	2	6	0	0	0	0	0	0	No scan			0	0	0
			15%			15%														
4	77	F	0	0	1	0	0	1	0	0	0	0	0	0	0	0	0	0	0	0
			2,5%			2,5%														
5	55	F	0	0	2	0	0	2	0	0	0	0	0	0	0	0	0	0	0	0
			5%			5%														
6	56	F	0	0	3	0	0	3	No scan			0	0	0	0	0	0	0	0	0
			7,5%			7,5%														
7	49	F	0	0	1	0	0	1	0	0	1	0	0	1	0	0	0	0	0	0
			2,5%			2,5%			2,5%			2,5%								
8	69	F	0	5	13	0	5	13	0	5	13									
			42,5%			42,5%			42,5%											
9	40	M	0	3	10	0	3	10	0	1	0	0	0	0	0	0	0	No scan		
			35%			35%			12,5%											
10	75	F	0	1	2	0	1	2	0	1	1	0	0	0	0	0	0	0	0	0
			7,5%			7,5%			7,5%											
11	51	M	1	6	20	1	6	20	1	6	15	0	2	10	0	2	8	0	2	8
			75%			75%			50%			25%			25%			25%		
12	64	M	0	5	10	0	5	10	0	2	3	0	1	0	0	0	0	0	0	0
			42,5%			42,5%			20%			12,5%								
13	39	F	0	1	3	0	1	3	0	0	0	0	0	0	0	0	0	0	0	0
			15%			15%														
14	32	F	0	1	3	0	1	3	0	0	0	0	0	0	0	0	0	0	0	0
			12,5%			12,5%														
15	65	F	0	1	4	0	1	2	0	0	0	0	0	0	0	0	0	0	0	0
			12,5%			7,5%														
16	71	F	0	5	12	0	5	11	0	0	0	0	0	0	0	0	0	0	0	0
			42,5%			42,5%														
17	56	M	0	3	6	0	3	6	0	0	1	0	0	0	0	0	0	0	0	0
			27,5%			27,5%			2,5%											
18	70	F	1	1	6	1	1	6	1	1	4	0	0	2	No scan			0	0	0
			30%			30%			15%			5%							0	

Gray = visible emboli. Black = deceased.

CTPA, Computed Tomography Pulmonary Angiography; MRI, Magnetic Resonance Imaging; QS, Qanadli score; Cen, Central; Lob, Lobar; Seg, Segmental.

The mean time between the CTPA and the first MRI was 18 h 32 min ± 9 h 34 min (median = 22 h 47 min). The time intervals for the MRI scans are shown in Table 3.

**Table 3.** The time between CTPA and subsequent MRI scans.

	First MRI	MRI 1 week (days)	MRI 1 month (days)	MRI 3 months (days)	MRI 6 month (days)
Mean	18 h 32 min	8.5	31.6	103.6	186.9
Median	22 h 47 min	7.8	32.3	97.9	185.7
SD	9 h 34 min	1.9	5.4	13.9	5.8

CTPA, computed tomography pulmonary angiography; MRI, magnetic resonance imaging.

All the MRI scans were of diagnostic quality.

Full resolution of the thrombi was observed in 0/18 (0%) patients at baseline (36 h), in 7/18 (39%) after one week, in 12/17 (67%) after one month, in 15/17 (88%) after three months, and in 16/17 (94%) after six months of anticoagulation. There was only one case where the emboli were still visible at six months. This patient also had the most significant clot burden at the initial CTPA and the highest Qanadli score in this cohort, with a 75% occlusion. (Table 2).

In three patients, CTPA scans were performed due to patients’ worsening symptoms and suspected progress of their PE. The CTPA for one patient was performed after two months, and for the other two patients after three months. In all three patients, no emboli could be seen on MRI after one week, and the results of the above mentioned CTPA scans were also all negative for PE.

**Fig. 1.**
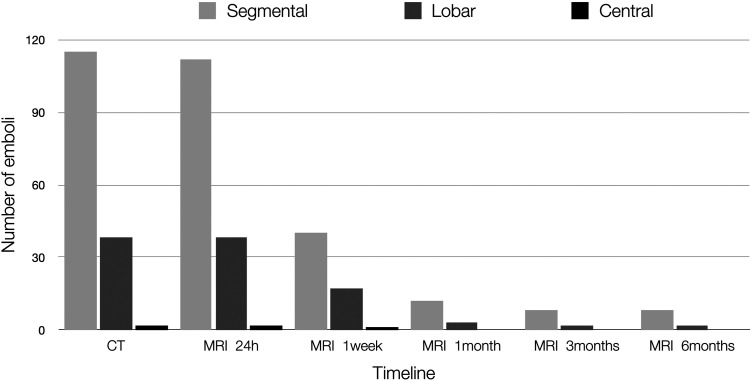
Remaining emboli at different levels.

The most significant resolution of the emboli happened within the first few weeks, with only 10% of the number of emboli seen at the first CTPA remaining at the one-month examination. The rate of resolution at the different levels was as follows: at the level of pulmonary arteries, a 50% decrease after one week and complete resolution after one month; at the level of lobar arteries, a 55% decrease after one week and only 8% remaining after one month; at the level of segmental arteries, a 63% decrease after one week and only 11% remaining after one month (Fig. 1).

We did not observe any movement of the emboli distally but, instead, we observed a gradual visual decrease in the size of the emboli at the original location until it was completely absent.

**Fig. 2.**
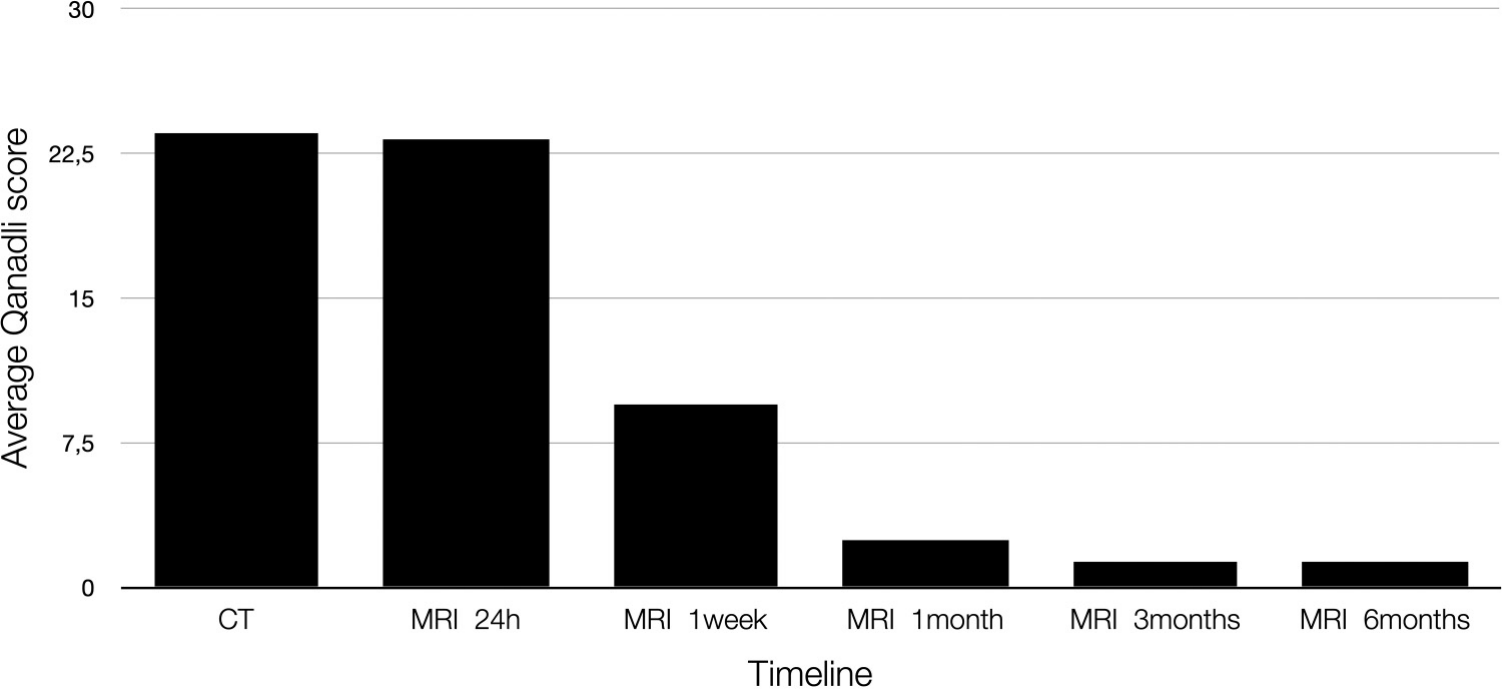
Development of the average Qanadli score with time.

The average Qanadli score was 23.6 at the diagnosis with CTPA and 23.3 at baseline MRI within 36 h, 9.4 after one week, 2.5 after one month, 1.4 after three months, and unchanged at 1.4 after six months. The scores for all the exams are shown in Table 2, and a graphical representation is shown in Fig. 2.

**Case 1:**
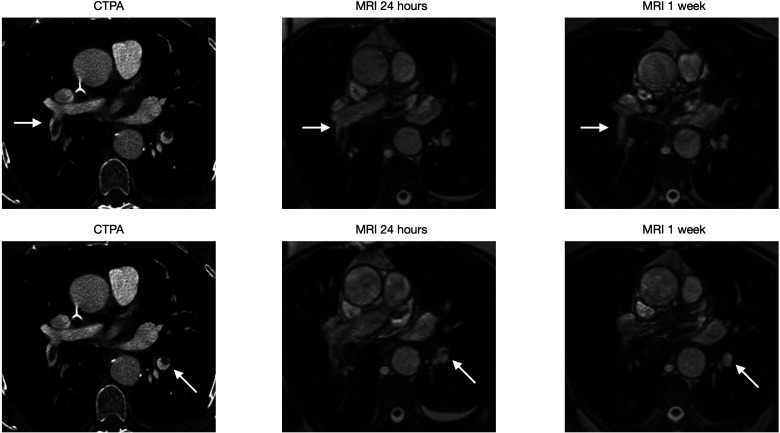
(Six images) A 71-year-old female with sigmoid cancer visited ER after one week of left-sided chest pain. CTPA showed multiple PE (arrow). PE at two different locations are seen and complete resolution is achieved in one week.

**Case 2a & 2b:**
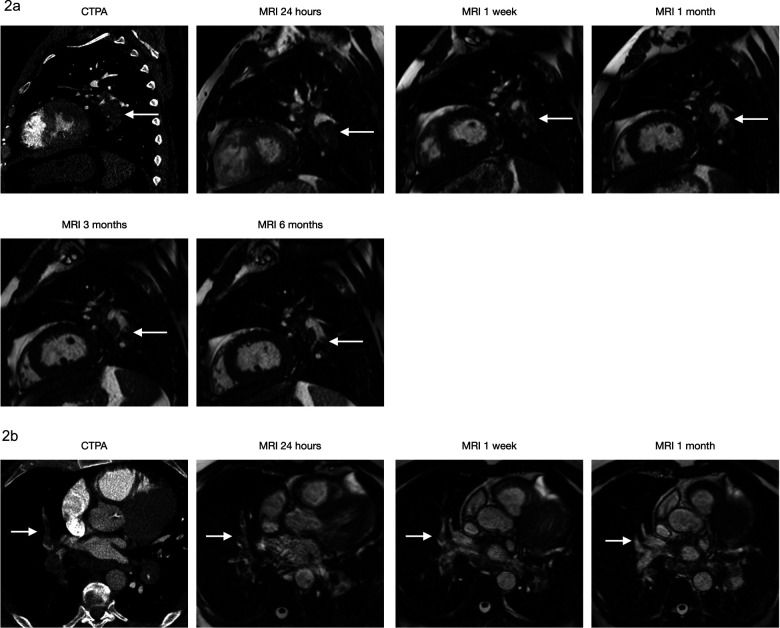
(10 images) A 71-year-old male visited ER after two weeks of cough and dyspnoea. His saturation at the time was 87%. CTPA shows massive bilateral PE with a Qanadli score of 75%. The 2a series shows PE (arrow) at the left lower lobe level, which decreases in size at follow up MRIs until one month. No significant change is observed after that. The 2b series shows PE (arrow) at the level of the left middle lobe, which decreases in size and has a complete resolution at one month.

**Case 3:**
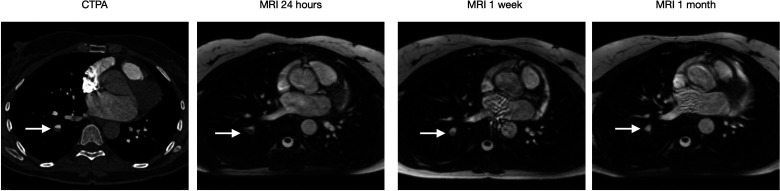
(Four images) A 49-year-old female visited ER after one week of dyspnoea and chest pain. CTPA showed PE (arrow) at the lower right lobe level. MRI shows a complete resolution at one month.

**Case 4:**
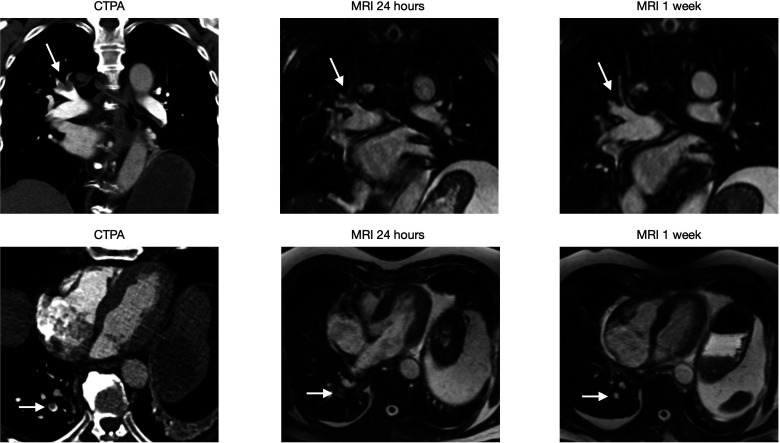
(Six images) A 77-year-old male visited ER after five days of chest pain. CTPA showed multiple right-sided PE (arrow). PEs at two different locations, both of which have a complete resolution at one week.

**Case 5:**
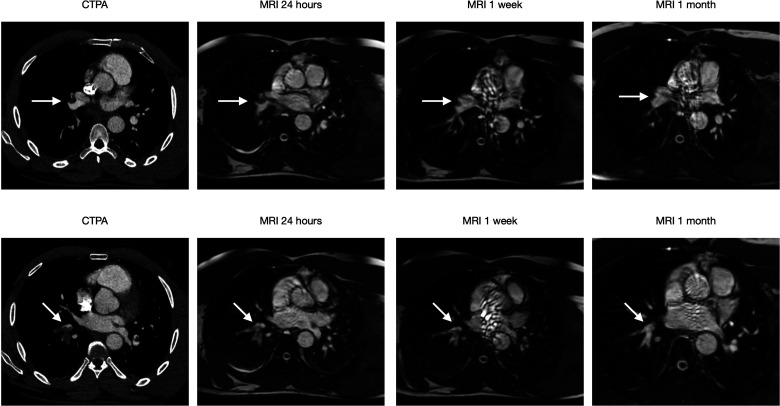
(Eight images) A 40-year-old male with sarcoma visited ER due to acute chest pain. CTPA showed multiple bilateral PE (arrow). PEs at two different locations, both of which decrease in size at the one-week exam and have a complete resolution at one month.

## Discussion

The principal finding of our study was the feasibility of monitoring the resolution of emboli over time using a non-contrast-enhanced, ungated MRI sequence. Emboli were found to remain in place, gradually shrinking until no longer visible with no signs of dislocation.

Previous studies reported in the literature for the follow-up of patients with PE have used either pulmonary angiography, CTPA, or V/Q scintigraphy (17–20), primarily based on retrospective analyses of patients who have had additional imaging due to worsening and/or residual symptoms after treatment. Due to the concerns of risk associated with radiation exposure, prospective studies using these modalities would be ethically challenging in most groups of patients, except for those in palliative care where future developments of a new malignancy would not be an issue. Previous retrospective studies often involve irregular intervals since they are not predetermined, and range from one day to a few years, making it difficult to follow the changes at the patient level (17–19,21). The available prospective studies usually have only one follow-up examination, ranging from three weeks to one year, making it impossible to compare the results of the present study where scans were made across several intervals (20,22,23).

The present method, which uses MRI as an alternative to scans involving radiation, allows multiple examinations in a predetermined schedule during the study's timeframe. The unenhanced, ungated MRI protocol has been published previously by Nyren et al. (12) and has been used occasionally in the last 10 years at our hospital to make a clinical decision when CTPA would pose a problem (13). The method is easy for the radiologist to read and does not differ greatly from reading CTPAs, as shown by Rogberg et al. (14) who evaluated the ability of residents to read these exams. This method enabled us to perform one CTPA and five MRI scans over a period of six months for each patient, which is unique for a follow-up study of PE.

We have compared our results with those of existing CTPA studies, as our method also provides us with the possibility of a direct visual comparison of the emboli. Follow-up studies using V/Q scans were not examined due to documented discrepancies between CTPA and V/Q scans (20,24).

In our study, the most significant resolution of the emboli was observed in the first week, with complete resolution in 39% of patients. That number increased to 67% at one month, and 88% at three months. This is in line with other prospective studies: for example, van Es et al. (20) presented a complete resolution in 41% (116/264) of patients after three weeks, while Extner et al. (23) saw complete resolution in 84% (132/157) of patients after six months. Similar results are also valid for retrospective studies that we have seen. For example, Choi et al. (25) showed a complete resolution in 24% (16/66) of patients at 3–7 days, 47% (42/90) at 8–21 days and 78% (121/155) at 22–90 days. The same applies to Stein et al. (17), who showed complete resolution in 40% (6/15) of the patients at 2–7 days and 81% (17/21) of patients at days 29–290. This timing of resolution has also been observed in a follow-up study of deep venous thrombosis by Haenen et al. (26).

However, a study by Remy-Jardin et al. (27) showed a complete resolution of only 48% (30/62) of patients, with a mean follow-up time of 10.5 months. Similarly, a study by Van Rossum et al. (28) showed complete resolution in only 32% (6/19) of patients between 4–9 weeks (mean = 5.6 weeks); all of the patients who did not show complete resolution were classified as having moderate or severe PE. In both cases, resolution rates are much lower compared to our findings and other studies discussed here. An explanation could be the higher prevalence of massive PE, particularly in the study by Remy-Jardin et al. (27), which included patients referred to the cardiology intensive care unit. The lower resolution rates would then be consistent with the fact that a high clot burden is known as a risk factor for the lower resolution rate of PE (29).

Regarding the decrease in the number of individual emboli, the present study found a reduction of 61% at one week, 90% at one month, and 93% at three months. The most significant percent decrease was observed at the segmental level, followed by the lobar and central levels. When examining complete resolution in those vessels, we see the reverse order with the central emboli resolving the fastest, which is in line with the results presented by Stein et al. (17). We also measured the clot burden using the Qanadli score, which showed a decrease of 59% after one week, 89% at one month, and 94% at three months. We did not find any correlation between the patients’ resolution rate versus predisposing risk factors for developing PE.

The presents study has some limitations. The most important factor is the low number of participants (n = 18). Another important aspect is that we did not follow the patients’ changes in symptoms and laboratory values at every MRI examination. Instead, we relied on information from the EMR, which is not particularly consistent across different hospitals; this is something we will address in future studies.

In conclusion, the present study serves as proof of concept for the use of MRI in tracking emboli. We found that the most significant resolution of PE occurred within the first few weeks of treatment, and in this cohort, all emboli decreased in size without changing location. The ability to visualize PE without radiation allows multiple examinations to be made without causing risk to the patient. This opens the potential for this MRI technique to be used in studies investigating clinical resolution or those evaluating the effect of drugs in clinical trials. It could also be used to evaluate incidence of chronic PE in future studies.
