# Erratum to “Feasibility of monitoring the resolution of acute pulmonary embolism with non-contrast-enhanced magnetic resonance imaging at one day, one week, one, three, and six months”

**DOI:** 10.1177/02841851221141156

**Published:** 2022-12-03

Medson K, Westerlund E, Paris RV, et al. Feasibility of monitoring the resolution of acute pulmonary embolism with non-contrast-enhanced magnetic resonance imaging at one day, one week, one, three, and six months. *Acta Radiol* 2022. EPub ahead of print September 14, 2022. DOI: 10.1177/02841851221122449

In the above mentioned article, the images of five cases that are described at the end of the article were inadvertently removed. The images of the five cases have been added to the article.

